# Stratification Of LIver Disease (SOLID): protocol for a prospective observational cohort study to determine the optimum biomarker strategies for the detection of advanced liver disease at the primary–secondary care interface

**DOI:** 10.1136/bmjgast-2022-001092

**Published:** 2023-02-08

**Authors:** Stuart McPherson, Helen Jarvis, John McGonigle, Joan Bedlington, Jill Dean, Kate Hallsworth, Elodie Hanon, Trevor Liddle, Ahai Luvai, Dina Mansour, Preya Patel, Laura Renwick, Dawn Teare, Christina Tanney, Quentin Anstee

**Affiliations:** 1 Liver Unit, Newcastle upon Tyne Hospitals NHS Foundation Trust, Newcastle upon Tyne, UK; 2 Translational & Clinical Research Institute, University of Newcastle upon Tyne, Newcastle upon Tyne, UK; 3 Newcastle NIHR Biomedical Research Centre, The Newcastle upon Tyne Hospitals NHS Foundation Trust, Newcastle upon Tyne, UK; 4 Population Health Sciences Institute, Newcastle University, Newcastle upon Tyne, UK; 5 Cruddas Park and Hillsview Surgery, Newcastle upon Tyne, UK; 6 LIVErNORTH Charity, Newcastle upon Tyne, UK; 7 Clinical Research Network North East and Cumbria, Newcastle upon Tyne, UK; 8 Department of Blood Sciences, The Newcastle upon Tyne Hospitals NHS Foundation Trust, Newcastle upon Tyne, UK; 9 Clinical Research Informatics, The Newcastle upon Tyne Hospitals NHS Foundation Trust, Newcastle upon Tyne, UK; 10 Gastrointestinal and Liver Services, Gateshead Health NHS Foundation Trust, Gateshead, UK

**Keywords:** fatty liver, cirrhosis, primary care, diabetes mellitus, alcohol

## Abstract

**Introduction:**

Undiagnosed fatty liver disease is prevalent in the community, due to high rates of harmful alcohol consumption and/or obesity. Fatty liver disease can progress to cirrhosis and its complications. Early identification of liver disease and treatment may prevent progression to cirrhosis. Biomarkers including FIB-4, enhanced liver fibrosis (ELF), PRO-C3 and vibration controlled transient elastography (VCTE) can stage liver fibrosis, but it is not known how well they perform in a primary care population. Moreover, no assessment of long-term prognostic ability of these biomarkers has been conducted in primary care. We aim to evaluate the performance of fibrosis biomarkers in primary care to develop a pathway to detect advanced fibrosis.

**Methods and analysis:**

This prospective, observational cohort study will recruit 3000 individuals with fatty liver disease risk factors (obesity, type 2 diabetes or hazardous alcohol consumption) at their primary care ‘annual chronic disease review’. Participants will have a ‘liver health check’. Two pathways will be evaluated: (1) all have FIB-4, ELF and VCTE performed, and (2) patients have an initial assessment with FIB-4 and ELF, followed by VCTE in only those with increased FIB-4 and/or ELF. Individuals with suspected significant/advanced liver fibrosis (liver stiffness measurement>8 kPa), will be reviewed in secondary care to confirm their fibrosis stage and institute treatment. The performance of FIB-4, ELF, PRO-C3, VCTE and novel biomarkers alone or in combination for advanced fibrosis/cirrhosis will be evaluated. Participants will be followed longitudinally via their electronic health records to assess long-term clinical outcomes.

**Ethics and dissemination:**

Ethical approval was obtained from the London-Chelsea Research Ethics Committee (22/PR/0535; 27 June 2022). Recruitment began on 31 October 2022. Outcomes of this study will be published in peer-reviewed journals and presented at scientific meetings. A lay summary of the results will be available for study participants and will be disseminated widely by LIVErNORTH.

**What is already known on this topic**

- Undiagnosed fatty liver disease is highly prevalent in the community, primarily due to high rates of alcohol consumption and obesity. The optimum approach to identify individuals with advanced fibrosis in the community remains unknown.

**What this study adds**

- This study will evaluate the performance of current and novel biomarkers to detect advanced liver fibrosis among patients at increased risk of fatty liver disease in primary care to determine the most clinically and cost-effective approach.

**How might the study affect research, practice or policy**

- This study will evaluate the impact of a liver fibrosis detection pathway in primary care and develop the most clinically and cost-effective pathway for widespread implementation or further study in a large randomised trial.

## Introduction

Undiagnosed fatty liver disease is highly prevalent in the community, primarily due to high rates of harmful alcohol consumption and/or obesity.^1^ Fatty liver disease can progress to cirrhosis and complications including liver failure and hepatocellular carcinoma. An estimated 1-in-5 individuals in the UK have non-alcohol-related fatty liver disease (NAFLD).^2^ Moreover, the prevalence of potentially harmful alcohol consumption is high in the community (15% females and 25% males in England) and alcohol accounts for 75% of deaths due to liver disease, costing the National Health Service (NHS) £3.5 billion per annum.^1^ The increasing prevalence of liver disease risk factors in the community and lack of recognition of liver disease are likely causes for the rising rates of cirrhosis and liver-related deaths.^1^

Because liver disease is usually asymptomatic at early stages, patients frequently present once end-stage liver disease has developed. One study showed that 73% of patients presenting with their first admission with cirrhosis or liver failure had never been referred to a liver clinic, which indicates a lack of early detection of liver disease.^1^ Importantly, most liver-related complications, mortality, healthcare costs and reduction in quality of life occurs in patients with cirrhosis rather than earlier stages of liver disease.^3 4^ Therefore, early identification of liver disease before cirrhosis and initiation of lifestyle changes may prevent progression to cirrhosis for some patients.^5^

Liver enzymes are often used in primary care to identify individuals with liver disease,^6^ but this approach is insensitive because up to 50% of patients with advanced liver fibrosis/cirrhosis have normal liver enzymes.^7^ Because stage of liver fibrosis is the key prognostic factor in liver disease,^8^ use of a test for fibrosis is likely to be far more efficient to identify advanced fibrosis. Therefore, taking a different approach and performing case finding using non-invasive tests to identify advanced liver fibrosis in individuals with liver disease risk factors may be an effective strategy.^9 10^

Numerous biomarkers have been proposed for the non-invasive staging of liver fibrosis.^11^ These include ‘indirect’ biomarkers, such as FIB-4 (age, AST, ALT and platelets),^12 13^ and ‘direct’ serum biomarkers that measure collagen matrix components, such as enhanced liver fibrosis ‘ELF’ test (hyaluronic acid, TIMP1 and P3NP)^14 15^ and more recently the novel collagen neo-epitope biomarker PRO-C3.^16 17^ In addition, vibration controlled transient elastography (VCTE; Fibroscan), an ultrasound-based technique that measures liver elasticity as a surrogate for fibrosis, has emerged as an effective non-invasive fibrosis test.^18^ As well as providing an accurate assessment of stage of fibrosis, these biomarkers can stratify patients for risk of liver-related events with reasonable accuracy.^19 20^

Current fibrosis biomarkers have advantages and disadvantages but the majority of data describing their performance is derived from secondary/tertiary care settings in patients with known liver disease (mainly NAFLD)^21^ and these tests have not been systematically evaluated in an unselected primary care population where their performance may be different. Therefore, an evaluation of their performance in a community ‘case finding’ setting is warranted. The FIB-4 score, which is effectively cost free, has been extensively evaluated in patients with NAFLD and can reliably exclude advanced fibrosis,^13 21^ but has a relatively high false positive rate for advanced fibrosis, particularly when used in individuals over the age of 65 years.^22^ Given this high false positive rate, a second-line confirmatory test is required.^6^ ELF has been evaluated in several liver diseases, including NAFLD and alcohol-related liver disease (ARLD).^23^ It performs reasonably well^15^ and has been used in a two-step fibrosis staging pathway after the FIB-4 for patients with raised liver enzymes in primary care, significantly improving the correct identification of advanced fibrosis compared with standard care.^24^ PRO-C3, the newest biomarker, has good performance for advanced fibrosis in NAFLD and ARLD.^16 17 25^ VCTE is now well established for staging liver fibrosis in patients with NAFLD and ARLD.^18 26 27^ Overall, a liver stiffness measurement (LSM) of <8 kPa reliably excludes advanced fibrosis/cirrhosis in patients with NAFLD and ARLD (sensitivity 93% and 94%, respectively), while an LSM of >12 kPa has 88% specificity for advanced fibrosis/cirrhosis in both ARLD and NAFLD.^26^ However, VCTE usually requires a specific attendance at a healthcare facility and needs to be performed by a trained operator, which could limit its use as a screening tool in primary care. VCTE also has a failure rate of approximately 5%, particularly among older patients (>60 years) and those with a Body Mass Index (BMI)>35 kg/m^2^.^28^

Recent evidence suggests that the diagnostic accuracy of blood fibrosis biomarkers may be different in a primary care population where the prevalence of advanced fibrosis is lower.^15 29^ Moreover, evaluation of the performance of liver fibrosis biomarkers has been cross-sectional and no assessment of long-term follow-up has been conducted to determine whether cases of advanced liver disease have been missed. Therefore, large-scale baseline and longitudinal assessment of the use of VCTE alongside other blood biomarkers to identify patients with advanced fibrosis in primary care is warranted.

## Aim

The overall aim of this study is to develop an effective pathway to identify patients with advanced liver fibrosis in individuals with risk factors for liver disease (obesity, type 2 diabetes (T2DM) or hazardous alcohol use) in primary care. The study will incorporate a ‘liver health check’ within primary care annual chronic disease reviews to identify advanced liver fibrosis. This pathway will be a platform to evaluate the performance of liver fibrosis biomarkers to develop the most clinically and cost-effective pathway for the early identification of advanced liver fibrosis in the community.

### Specific aims

1. To implement and assess the effectiveness of primary care-based risk-stratification pathways using VCTE and blood biomarkers to identify advanced liver fibrosis and predict outcomes among individuals with liver disease risk factors.
2. To evaluate whether implementation of the pathways increases the diagnosis of patients with advanced fibrosis compared with standard care.
3. To use the Stratification Of LIver Disease (SOLID) platform to assess the performance of novel and established fibrosis biomarkers and so determine the optimum biomarker strategy for wider implementation within the NHS.
4. To assess the cost effectiveness of novel and established risk stratification pathways.
5. To assess the acceptability of embedding the pathways into routine primary care chronic disease management to guide wider implementation.

## Methods and analysis

### Study design

SOLID is a prospective, observational cohort study where individuals with risk factors for fatty liver disease will have a liver health check to identify advanced liver fibrosis/cirrhosis when they attend their primary care annual chronic disease review. The diagnostic performance of fibrosis biomarkers for advanced fibrosis/cirrhosis with respect to long-term outcomes will be evaluated.

**Figure 1:**
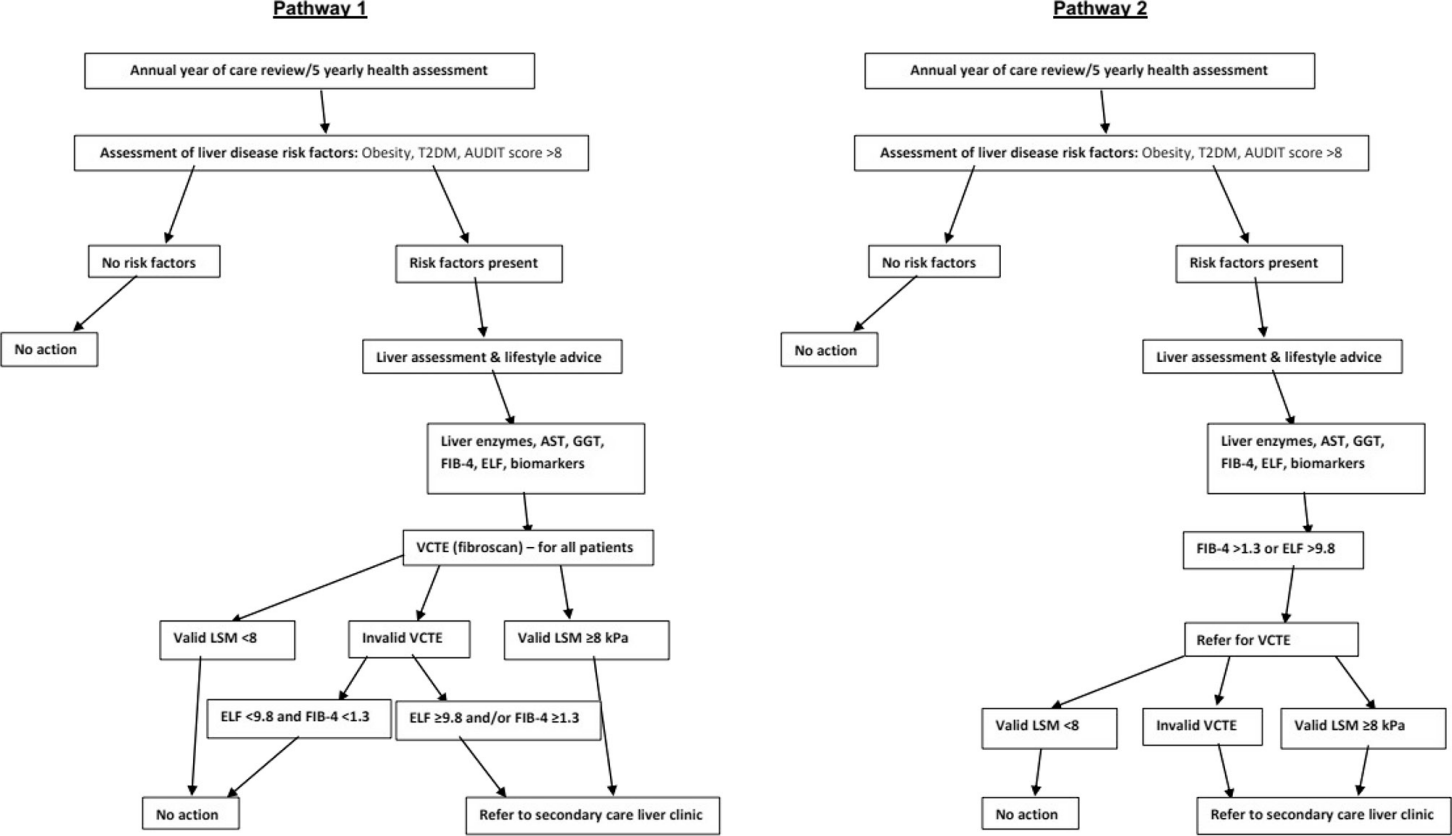
An overview of the clinical assessment pathways for liver fibrosis in primary care. ALP, alkaline phosphatase; ALT, alanine aminotransferase; AST, aspartate aminotransferase; AUDIT, Alcohol Use Disorders Identification Tool; ELF, enhanced liver fibrosis test; GGT, gamma glutamyl transferase; LSM, liver stiffness measurement; T2DM, type 2 diabetes; VTCE, vibration controlled transient elastography.

Primary care annual chronic disease reviews offer an ideal place to conduct a liver risk assessment since much of the information needed for this is already collected. Liver risk factors will be assessed, and individuals with obesity (BMI>30 kg/m^2^), T2DM or hazardous alcohol consumption (using Alcohol Use Disorders Identification Tool; (AUDIT score>8)) will have a ‘liver health check’ using established fibrosis biomarkers including FIB-4, ELF and VCTE. Blood samples will be taken for novel biomarkers, including PRO-C3, to evaluate their performance. Two pathways will be evaluated (figure 1): (1) all patients have FIB-4, ELF test and VCTE performed, and (2) patients have an initial assessment with the FIB-4 and ELF test, followed by VCTE in only those with increased FIB-4 and/or ELF.

Irrespective of which pathway a patient is recruited into, individuals with suspected moderate or advanced liver fibrosis (LSM>8 kPa), will be reviewed in secondary care to confirm their stage of liver fibrosis and institute specific treatment and/or enhanced lifestyle management depending on the underlying diagnosis within routine NHS clinical services.^30 31^ When cirrhosis is identified, patients will be entered into liver cancer and varices surveillance programmes according to current standard of care.^32^

These pathways will allow us to evaluate the diagnostic accuracy of FIB-4, ELF, PRO-C3, VCTE and novel biomarkers alone or in combination for advanced fibrosis/cirrhosis in individuals with risk factors for liver disease. Unique to this study, participants will also be followed longitudinally via their electronic health records and NHS Digital to assess long-term clinical outcomes.

### Study participants

We aim to recruit 3000 participants from primary care networks in NE England

### Inclusion criteria

Individuals aged 18–80 years attending for an ‘annual review of care’, ‘chronic disease review’ or ‘health check’ with one or more of the following risk factors:

1. Obesity (BMI>30 kg/m^2^).
2. T2DM.
3. Potentially hazardous/harmful alcohol consumption (AUDIT score>8).^33^

### Exclusion criteria

1. Life limiting disease on high risk or palliative care register.
2. Known liver disease under secondary care follow-up.
3. Lack of capacity to provide informed consent
4. Patient unable to understand or speak English.

### Study outcomes

#### Primary outcome

The primary outcome of SOLID is to assess the number of patients identified with advanced liver fibrosis/cirrhosis using each of the pathways based on an overall clinical evaluation by a panel of hepatologists.

The presence of advanced fibrosis will be determined by an overall clinical assessment after secondary care review and will include all investigations undertaken in primary and secondary care. Where there is uncertainty about the stage of liver fibrosis, a liver biopsy will be offered as per usual clinical care. The overall clinical assessment for advanced fibrosis will be undertaken by three hepatologists. Where cases are equivocal, all the clinical data for these patients will be reviewed independently by each of the hepatologists to determine the presence of advanced fibrosis. An agreement analysis will be conducted to determine the concordance between hepatologists. Discrepancies will be resolved by consensus.

#### Secondary outcomes

1. Assess the utility of non-invasive testing/triage strategies to identify patients who have liver-related events (death, hepatic decompensation and transplantation) during longitudinal follow-up at 2, 5 and 10 years via electronic care records/NHS Digital.
2. Compare the number of new cases of advanced fibrosis/cirrhosis identified with previous years in participating practices and control practices historically and over the study period.
3. Assess the performance of FIB-4, ELF, PRO-C3 and other novel blood-based biomarkers alone or in combination to identify advanced fibrosis/cirrhosis as defined by overall clinical assessment or LSM.
4. Assess the performance of the biomarkers to correctly exclude advanced fibrosis/cirrhosis.
5. Assess the uptake of the liver assessment, attendance rates for VCTE and liver clinic appointments with both pathways.
6. Conduct a subsequent cost effectiveness analysis of established and novel pathways.
7. Assess barriers and facilitators to incorporating the pathway into routine primary care practice.
8. Development of the optimum pathway (clinically and cost-effective and implementable) for further evaluation.

### Recruitment and consent

Potential participants will be identified prior to their review appointment. An invitation to the study and Participant Information Sheet (PIS) will be sent via post, email or text prior to their appointment as per the practice’s usual procedure.

The study will use electronic remote consent. Within their invitation letter, patients will be given log in details for the study consent portal where they can give consent and then input some clinical details to assess their eligibility for the study (current weight and height to determine BMI, known history of T2DM and AUDIT score^33^). A member of the research team will support individuals to complete the consent process. Patients who meet the eligibility criteria and give consent will then be asked to complete some data collection fields (eg, ethnicity, alcohol consumption, smoking history, EQ-5D-L^34^). When patients attend for their review appointment, study staff will confirm consent (verbally and document in the electronic patient record). Study procedures (table 1) will then be performed at the annual review.

**Table 1:** Study procedures

Clinical data collected	Blood samples
Date of birth	FBC
Age at time of assessment	Liver enzymes (albumin, bilirubin, ALT, ALP)
Sex at birth	AST
Ethnicity	GGT
Weight	HbA1c
Height	FIB-4 score
BMI	Lipids
Waist and Hip circumference	ELF
Blood pressure	PRO-C3
AUDIT score^33^	Blood for storage to assess future novel biomarkers of liver disease
Current alcohol consumption (average units/week over last year)	
History of previous heavy alcohol consumption (>35 U/week for females or >50 U/week for males) for >1 year	
Smoking history	
Relevant medical history including: hypertension, T2DM, dyslipidaemia, obstructive sleep apnoea	
Current medications	
EQ-5D-L questionnaire^34^	

ALP, alkaline phosphatase; ALT, alanine aminotransferase; AST, aspartate aminotransferase; AUDIT, Alcohol Use Disorders Identification Tool; ELF, enhanced liver fibrosis test; EQ-5D-L, EuroQol-5 Dimensions-Level; FBC, full blood count; GGT, gamma glutamyl transferase; T2DM, type 2 diabetes.

For individuals who would like to take part but do not want to use remote electronic consent, informed consent discussions will be undertaken by trained staff in accordance with good clinical practice (GCP) and study procedures performed at the annual review.

A screening log will be kept to document details of subjects invited to participate in the study. This will be to CONSORT (CONsolidated Standards Of Reporting Trials) recommendations to ensure data available within the observational cohort are not influenced by unexpected bias in those declining participation. The log will also ensure that potential participants are only approached once.

### Study procedures

All participants will have a liver fibrosis assessment using established biomarkers (VCTE and/or FIB-4 and ELF), blood tests and relevant clinical and demographic data collected at the time of assessment, as shown in table 1.

The optimum clinical pathway for the evaluation of liver fibrosis is not known so we plan to evaluate two pathways (figure 1) to determine which is most clinically and cost-effective. In pathway one, all patients will be offered VCTE and have blood tests for liver fibrosis biomarkers. In pathway two, patients with FIB-4>1.3 and/or ELF>9.8 indicating possible advanced fibrosis/cirrhosis will have VCTE to stage fibrosis.

VCTE will be conducted by trained operators at Newcastle Hospitals or in the primary care centre depending on the proximity of primary care sites to the hospital (sites close to the hospital will have VCTE performed at the hospital and those further away will have it performed at the practice). LSMs and controlled attenuation parameter readings will be obtained using a Fibroscan 430 mini+machine with M or XL probe as per the manufacturer’s recommendations. VCTE reading will be considered ‘reliable’ if they meet the accepted quality criteria of at least 10 readings from the same location with a single probe and the IQR/median ratio is less than 30% when the LSM exceeds 7.1 kPa.^35^

Individuals will be given a report of their liver assessment (LSM or FIB-4/ELF) to indicate their stage of liver fibrosis. All participants will be given relevant lifestyle advice and an information booklet entitled ‘Looking after your Liver’ (online supplemental file 1).

10.1136/bmjgast-2022-001092.supp1 Supplementary data

Individuals with an LSM<8 kPa or who have low FIB-4/ELF (pathway 2), which excludes advanced fibrosis, will remain in primary care with further monitoring as per their usual care. Individuals with an LSM>8 kPa, indicating possible moderate to advanced liver fibrosis, will be referred to secondary care for further assessment and treatment. VCTE has a failure rate of approximately 5%. For individuals where it is not possible to obtain a reliable LSM, the ELF test and FIB-4 will be used as an alternative test to assess fibrosis. Both the FIB-4 and ELF tests can reliably exclude advanced fibrosis in patients with fatty liver disease.^13 14^ Individuals with a low ELF (<9.8) and FIB-4 score (<1.3) will be managed in primary care, while those with an ELF>9.8 and/or FIB-4 score>1.3 will be referred to secondary care for further assessment.

Individuals with raised liver blood tests will have investigations as per standard care following the Northeast and North Cumbria Abnormal Liver Blood Test Guidelines.^36^

### Secondary care assessment for individuals with suspected moderate/advanced liver fibrosis

Investigations and management in secondary care will be conducted as per usual clinical practice (including clinical history and examination, blood liver aetiology screen, liver ultrasound and liver biopsy or other imaging, where appropriate).^30 31^ Management will depend on the underlying diagnosis and stage of liver fibrosis. Relevant clinical data will be collected from the patients’ clinical encounters in secondary care including:

1. Final stage of liver fibrosis determined by overall clinical assessment using all available results (primary endpoint).
2. Secondary care diagnosis of cause of liver disease.
3. Details of further investigations carried out as per usual clinical care (blood tests, imaging, liver biopsy).
4. Relevant medical history and medications.
5. Health-related quality of life questionnaire (CLDQ, Chronic Liver Disease Questionnaire).^37^

### Proposed analyses in the study

#### Description of the cohort

The baseline characteristics of the cohort and primary and secondary outcomes will be described using descriptive statistics.

#### Assessment of the performance of the biomarkers

Diagnostic accuracy of the biomarkers will be assessed using the primary and secondary endpoint reference standards. The performance of the non-invasive tests to identify advanced fibrosis will be assessed by receiver operating characteristic curve analysis. The sensitivity, specificity, positive predictive values, negative predictive values, positive likelihood ratios (LR+ve) and negative likelihood ratios (LR−ve) will be calculated for each test or combination of tests using established cut-offs. A number of post hoc analyses will be conducted including the assessment of the performance of age specific cut-offs for the FIB-4 score to diagnose/exclude advanced fibrosis^22^ and a review of the performance of the biomarkers in subsets of patients with suspected NAFLD, ARLD and metabolic dysfunction associated fatty liver disease.^38^ Moreover, we will assess the impact of combinations of liver disease risk factors and other metabolic risks (eg, pre-diabetes, hypertension and dyslipidaemia) in identifying advanced liver disease. The clinical efficacy of pathways to detect advanced fibrosis using the biomarkers alone or in combination will be modelled using the data obtained from the study to develop optimal pathways.

#### Sample size calculation and power estimates

This study will recruit 3000 participants from Primary Care Networks in Northeast England. Searches in two practices indicate that at least 2600 of 20 000 patients meet the eligibility criteria and attend annual year of care reviews. In addition, approximately 200 patients attend 5-yearly health reviews. Approximately 1500 patients will be recruited into each pathway. We estimate that the prevalence of moderate/advanced fibrosis in the recruited population will be 5%–7.5%.^9 39 40^

With a prevalence of advanced fibrosis of 5%–7.5%, this will give us 150–225 true positives and 2775–2850 true negatives. The large sample of true negatives will result in higher precision to estimate specificity than sensitivity. The total sample size of 3000 will provide a precise estimate of the true prevalence for this population. If the true prevalence is 5%–7.5%, the expected width of the 95% CI for the population prevalence is 0.06–0.019.

#### Assessing the impact of the novel pathway on diagnosis rates of advanced liver fibrosis compared with usual care

To assess the impact of the pathways for identifying new cases of advanced fibrosis, a comparison of rates of identification of new cases of advanced fibrosis will be conducted in the participating practices in preceding years (avoiding COVID-19 as a confounder). Rates of advanced fibrosis will be determined by the number of patients (age 18–80 years) referred to secondary care gastroenterology/liver services in the period who received a diagnosis of advanced fibrosis/cirrhosis. Regional guidelines recommend that patients with suspected advanced fibrosis/cirrhosis are referred to secondary care,^36^ so this methodology should identify those new diagnoses.

The number of new diagnoses of advanced liver fibrosis/cirrhosis will be assessed in four control practices that have similar demographics and clinical management over the study period to determine rates of diagnosis of advanced fibrosis/cirrhosis diagnosis by current clinical pathways.

#### Long-term follow-up of patients

To date, studies assessing fibrosis biomarkers have not collected longitudinal data on their performance for hard endpoints such as liver-related events and mortality. In addition, they have not been able to determine the ‘miss’ rate for clinically significant liver disease. In order to assess the long-term prognostic ability of liver fibrosis biomarkers, long-term data will be collected, including liver-related endpoints and mortality (liver related and all-cause), on the patients from their electronic care records. Electronic patient records will be accessed via NHS Digital at intervals to assess long-term outcomes at 2, 5 and 10 years so that biomarker prognostic performance for prediction of liver-related events can be determined.

#### Mixed-method analysis of patient and clinician experience of the novel pathway for liver disease care

Pathway success will be quantified using the NoMAD survey instrument,^41^ developed and validated to measure the implementation success of complex interventions in healthcare from the perspective of involved healthcare professionals. Qualitative interviews with study patients, as well as healthcare professionals will be carried out to explore barriers and facilitators to successful implementation in more detail. The survey is based on, and the interviews will be guided by Normalisation Process Theory.^42^ This work will be conducted in a substudy led by Dr Helen Jarvis (IRAS ID 317792, REC reference 22/WA/0240).

#### Determining the cost-effectiveness of the liver fibrosis pathways

Data will be collected to undertake a subsequent cost-effectiveness analysis of the pathways with an initial model developed using results of the study and data assembled from the literature. A revised model incorporating results of the longer term follow-up will be subsequently developed. This will be undertaken as a separate substudy.

### Data collection and record keeping

Clinical data will be collected by appropriately GCP healthcare professionals in the participating sites. A standardised clinical data collection form will be used, which is embedded into the electronic health record (SystemOne or EMIS). All study data will be uploaded into a REDCap database. A standardised clinical data collection form will also be used for patients reviewed in secondary care and data uploaded to REDCap. REDCap is a secure database, fully compliant with GCP, EU and UK regulations, allowing a full audit trail for tracking data manipulation and user activity and is backed-up on a frequent basis. Standard data sharing agreements will be in place with individual GP practices allowing for upload of study data.

### Public and patient involvement

Input into the study design, generation of protocol, PISs and patient information booklets has been kindly provided by LIVErNORTH, a liver patient support group. Patient representatives are part of the SOLID Steering Committee.

### Ethics and dissemination

This study received favourable opinion from the London-Chelsea Research Ethics Committee on 27 June 2022 (REC reference 22/PR/0535; IRAS ID 310086) and Health Research Authority (HRA) approval was received on 11 July 2022. Recruitment began on 31 October 2022 and is planned to end on 31 August 2024.

Publication is the responsibility of the investigators. Authorship principles will follow the International Medical Editors conventions. The outcomes of this study will be published in peer-reviewed journals and presented at scientific meetings. A lay summary of the results will be available for study participants and will be disseminated widely by LIVErNORTH.

## Discussion

Undiagnosed fatty liver disease is prevalent in the community and as a result mortality rates from liver disease are rising.^1^ There is currently no widely recognised approach to detect liver disease in the community and several strategies have been suggested, but the optimum approach remains unknown.^10^ Therefore, the main aim of this study is to assess the efficacy of two pathways to detect advanced liver fibrosis among patients at increased risk of fatty liver disease in primary care. Data collected will allow us to evaluate the performance of current and novel liver fibrosis biomarkers in this setting and develop effective pathways for widespread implementation.

### Strengths

This is one of the first studies to systematically assess the performance of multiple liver fibrosis biomarkers in a large cohort of well-phenotyped participants from primary care. We are recruiting from a variety of primary care settings, including the inner city and rural areas to make the findings widely applicable. Crucially, in addition to assessing biomarker performance to detect advanced fibrosis at baseline, we are collecting data on long-term outcomes for up to 10 years to assess the prognostic ability of biomarkers for liver-related outcomes, which has never been assessed in this setting.

A major strength of this study is that we are running the study close to ‘real life’ to facilitate recruitment and enable widespread implementation after the study. The liver health checks are being conducted during established primary care chronic disease reviews and health checks, which is an efficient use of resources since much of the information required is already collected. Remote electronic consent will enable many of the research elements of the study (eg, questionnaires) to be conducted electronically prior to the annual review appointment, meaning healthcare assistants can focus on conducting the key clinical aspects like obtaining blood tests and taking anthropometric measurements. Many of the participants will require an additional appointment for VCTE to stage liver fibrosis as this is a key fibrosis endpoint for the study. The design of our study will allow us to determine whether VCTE can be used in a more targeted manner.

### Limitations

The main limitation of this study is the reference standard for advanced fibrosis. Currently, the standard used to define liver fibrosis stage in clinical trials is liver biopsy, but clearly it would be impossible to conduct liver biopsies in all patients in this study. We are therefore using an ‘overall clinical assessment’ to identify patients with advanced fibrosis, which will determined by three hepatologists and will incorporate all available investigations. Some patients may also have a liver biopsy conducted as per usual care if there is diagnostic uncertainty. Although imperfect, this methodology has been used effectively in another ‘real world’ study conducted in primary care.^24^
