# Supplementary material for: Stratification Of LIver Disease (SOLID): protocol for a prospective observational cohort study to determine the optimum biomarker strategies for the detection of advanced liver disease at the primary–secondary care interface
Source: BMJ Open Gastroenterol. 2023 Feb 8;10(1):e001092. doi: 10.1136/bmjgast-2022-001092 (PMC9923258; doi:10.1136/bmjgast-2022-001092)
Supplement: Supplementary data [file bmjgast-2022-001092supp001.pdf]

BMJ Publishing Group Limited (BMJ) disclaims all liability and r
placed on this supplemental material which has been supplied

5

LOOKING
AFTER
YOUR
LIVER

Looking After Your Liver

reducing your risk of fatty liver disease

NORTH

information leaflet 5
v1 April 2022

BMJ Publishing Group Limited (BMJ) disclaims all liability and r[illegible]
placed on this supplemental material which has been supplied [illegible]

Glossary of Terms used in this booklet:

Non-alcoholic fatty liver disease (NAFLD): build up of fat in the liver associated with being overweight or having type 2 diabetes.

Alcohol-related fatty liver disease: build up of fat in the liver due to drinking more than the recommended limits for alcohol.

Liver fibrosis: a build up of scarring in the liver

Cirrhosis: severe scarring (fibrosis) of the liver that can lead to reduced liver function or cause complications such as liver cancer or liver failure.

Body mass index (BMI): a measure of your weight relative to your height to give an indication of whether you have a healthy weight.

Notes:

BMJ Publishing Group Limited (BMJ) disclaims all liability and r[illegible]
placed on this supplemental material w[illegible] has been supplied

Looking after your liver – reducing your risk of fatty liver disease

This booklet aims to give you some advice to help reduce your risk of developing liver disease in the future. Fatty liver disease is very common and affects 1 in 4 adults in the UK. This condition is caused by a build-up of fat within the liver and most often occurs in people who are overweight, have diabetes or who consume more than the recommended limit for alcohol. Fatty liver disease is one of the major causes of cirrhosis (severe scarring of the liver). Importantly, fatty liver disease can be reversible in many people if they make lifestyle changes. Healthy eating, weight loss, increasing physical activity and exercise as well as reducing or stopping alcohol consumption can all help reduce fat in the liver.

What is fatty liver disease?

Non-alcohol-related fatty liver disease (NAFLD) is caused by a build-up of fat within the liver and can occur in people who are overweight, have diabetes or have high cholesterol. In this condition, fat droplets develop within the liver cells causing damage, which can lead to liver inflammation and scarring (fibrosis). Some people with NAFLD go on to develop cirrhosis. People with fatty liver might not know that they have the condition because it often doesn't cause any symptoms. Many people have undiagnosed NAFLD.

- 7 in 10 people with Type 2 diabetes have NAFLD
- 8 in 10 people with obesity have NAFLD
- 1 in 20 people with NAFLD develop progressive liver disease leading to cirrhosis

Alcohol-related fatty liver disease: Drinking more than the recommended limits for alcohol can also lead to a build-up of fat in the liver leading to liver inflammation, fibrosis and cirrhosis.

- Drinking more than14 units of alcohol a week increases the risk of liver disease and other alcohol-related complications such as cancer and dementia

BMJ Publishing Group Limited (BMJ) disclaims all liability and r
placed on this supplemental material which has been supplied

- Over 90% of heavy alcohol drinkers (more than 50 units a week for males or more than 35 units a week for females) will develop fatty liver and 25% of heavy drinkers will develop cirrhosis.

People with obesity who also drink more than the recommended limit for alcohol have the highest risk of developing fatty liver disease and cirrhosis

Why is it important to avoid developing fatty liver disease?

- One in 20 people with fatty liver will develop liver cirrhosis (severe scarring of the liver). People with cirrhosis are at risk of developing liver failure or liver cancer.
- Fatty liver disease increases the risk of developing diabetes, heart disease and stroke.

Can you reverse fatty liver disease?

Importantly, in many people fatty liver disease is reversible with lifestyle change, particularly with weight loss through healthy eating and increased physical activity:

- Weight loss of more than 3% body weight can reduce liver fat.
- Weight loss of more than 5% body weight can reduce liver inflammation.
- Weight loss of more than 10% body weight can reverse liver fibrosis.
- Exercise can reduce liver fat.

Reducing or stopping alcohol consumption can improve fatty liver disease, particularly in people who drink more than the recommended limits for alcohol. Alcohol also contains a lot of calories so reducing alcohol intake can help with weight loss as well.

BMJ Publishing Group Limited (BMJ) disclaims all liability and [illegible] placed on this supplemental material which has been supplied [illegible]

Given that cirrhosis can cause life threatening complications, it is important that fatty liver disease is identified and treated at an early stage before cirrhosis develops.

How do I look after my liver?

Eating a healthy diet

A well-balanced and healthy diet is essential to avoid damage to your liver. The following advice can help:

- Try to eat regular meals and avoid snacking in between.
- Control your calorie intake through eating smaller portions.
- Avoid foods that are high in sugar or saturated fat (avoid the red traffic light labelled food).
- Eat at least 5 portions of fruit and vegetables per day.
- Eat some beans, pulses, fish, eggs and lean meat or vegetarian protein.
- Avoid eating large amounts of carbohydrate-based foods such as potatoes, bread, rice or pasta – carbohydrate should only be about a third of what you eat each day.
- Drink 6-8 glasses of water per day – try to avoid sugar-sweetened drinks.

For more detailed dietary advice then look at the '**Eat well – NHS' website** (www.nhs.uk/live-well/eat-well/) or ask your GP, practice nurse or dietitian for some information.

Please note – if you are diabetic it is important you discuss this with your diabetes support team and dietitian. Control of your diabetes and blood sugars is essential in helping reversal of fatty liver disease.

Work out your body mass index (BMI)

Your BMI is a measure of your weight relative to your height and gives an indication of whether you have a healthy weight. You can calculate your BMI by putting

[illegible]l. BMJ Open Gastro 2023; 10:e001092. doi: 10.1136/bmjgast-2022-001092

BMJ Publishing Group Limited (BMJ) disclaims all liability and r placed on this supplemental material which has been supplied

your height and weight into an online calculator (www.nhs.uk/live-well/healthy-weight/BMI calculator/) or ask your GP, nurse or dietitian to calculate it for you

- Healthy weight = BMI less than 25 kg/m^2 (less than 23 kg/m^2 for Asian ethnic groups)
- Overweight = BMI 25-30 kg/m^2 (23-27.5 kg/m^2 for Asian ethnic groups)
- Obese = BMI more than 30 kg/m^2 (more than 27.5 kg/m^2 for Asian ethnic groups)

If you have a BMI in the overweight or obese category this increases the risk of developing fatty liver, diabetes and heart disease. In most cases weight loss would improve your health and reduce your risk of developing these weight-related conditions.

Some tips for successful weight loss

- Monitor and record your weight weekly to assess your progress.
- Set short-term realistic goals as well as having a longer-term goal.
- Aim to lose up to 0.5-1 kg (1-2 pounds) per week.
- Monitor what you are eating.
- Plan your meals to avoid being tempted by unhealthy foods or drinks in the shops.
- Increase the amount of physical activity you do each day.

Physical activity, exercise and your liver

Being more physically active can help reduce liver fat and improve your fatty liver disease. The more physically active you are, the better.

- Exercise has been shown to decrease liver fat independent of weight loss
- Increasing daily physical activity can help you to lose weight alongside changes in your diet
- Importantly: physical activity and exercise can help you to maintain weight loss in the long term

There are lots of other health benefits of increasing physical activity and exercise if you have fatty liver disease.

These include:

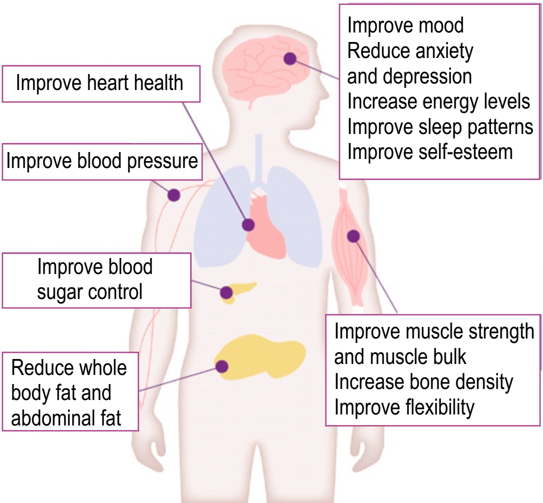

What type and how much physical activity/exercise do I need to do?

The NHS recommends doing two different types of activity - moderate aerobic exercise ('cardio'; walking, running, swimming or cycling) and strengthening exercises (lifting, pulling or pushing exercises). Both of these can help prevent or treat fatty liver disease.

Aim for at least **30 minutes of moderate intensity exercise a day on at least 5 days each week**. This can be broken down into 10-minute bouts, if this is more manageable. If you can't do the recommended amount at the moment, don't worry. Make a start by doing what you can. As your body gets used to being more active, build up your activity levels by pushing yourself a bit harder or keeping going for longer. You should feel a bit out of breath and warm. Do **strengthening exercises** that work all your major muscle groups (legs, hips, back, abdomen, chest, shoulders and arms) on **2 days a week**.

BMJ Publishing Group Limited (BMJ) disclaims all liability and r
placed on this supplemental material which has been supplied

It is important to set yourself an achievable goal and try to gradually build up to this – the more physical activity/exercise you can do, the better!

Ideas for increasing your activity levels

- Walk for part of the route to work (for example, get off the bus one stop earlier)
- Break up sitting time by standing up and walking for one minute every hour
- Take the stairs instead of the lift
- Walk the dog at least once a day
- Join a walking group
- Try a new exercise class
- Do some gardening or mow the lawn

F**or more detailed physical activity advice/ideas then visit the NHS 'How fit today' website** (www.howfittoday.co.uk) or
Sport England's 'We are undefeatable' website (https://weareundefeatable.co.uk/).
You can also get information from you GP or practice nurse.

Safer drinking

Alcohol consumption can contribute to the development of fatty liver if you drink more than the recommended limits for alcohol. It is therefore important to stay within the current guidelines for health.

- The maximum recommended alcohol limit for both men and women is 14 units per week. Details of the number of units in alcoholic drinks are shown below. To calculate the number of units of alcohol you drink each week use the online calculator at https://alcoholchange.org.uk/alcohol-facts/interactive-tools/unitcalculator
- Have at least two alcohol-free days every week and avoid binge drinking.

BMJ Publishing Group Limited (BMJ) disclaims all liability and r
placed on this supplemental material which has been supplied

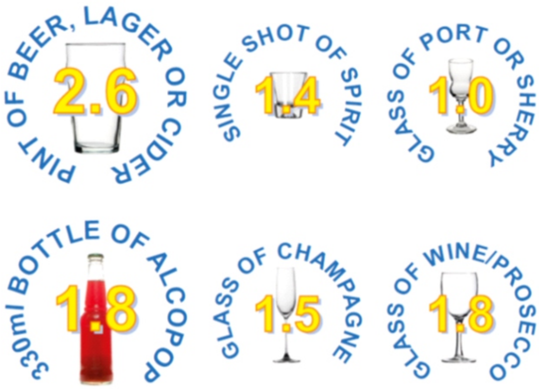

- If you have scarring of the liver or cirrhosis, alcohol may make the condition worse and it's advisable to stop drinking alcohol completely.
- If you drink alcohol every day and get withdrawal symptoms when you don't drink alcohol (shaking and symptoms of anxiety) then it is important that you don't stop drinking suddenly as you may be at risk of having an alcohol withdrawal fit. If you have these symptoms then you should seek advice from an alcohol specialist.

If you would like help to reduce your alcohol intake then you can get advice from https://alcoholchange.org.uk/help-and-support/get-help-now

or you can self-refer by contacting:

- **Newcastle Treatment and Recovery (NTAR)**

  0191 2061117
- **Northumberland Treatment and Recovery**

  01670 798 200
- **North Tyneside Treatment and Recovery**

  0191 2408122
- **Recovery Steps Cumbria**

  01900 512300

BMJ Publishing Group Limited (BMJ) disclaims all liability and [illegible]
placed on this supplemental material which has been supplied [illegible]

Smoking and fatty liver disease

Smoking is discouraged if you have fatty liver disease, as it can accelerate disease progression. Smoking also increases your risk of developing liver cancer, heart disease and lung disease so it is important for your general health to stop smoking if possible.

If you would like support to stop smoking then please contact:

- https://www.nhs.uk/live-well/quit-smoking/
- **Newcastle stop smoking helpline** *0191 269 1103*
- **Northumberland stop smoking helpline** *01670 813135*
- **Durham stop smoking helpline** *0191 369 2016*
- **Cumbria stop smoking helpline** *0300 013 3000*

Useful Contacts

For further information, you can contact LIVErNORTH - details overleaf and on the back cover.

Credits & references

This guide has been produced by Dr Stuart McPherson, Consultant Hepatologist at the Freeman Hospital, Newcastle upon Tyne with the collaboration of the LIVErNORTH National Liver Patient Charity.

Notes:

[illegible]l. BMJ Open Gastro 2023; 10:e001092. doi: 10.1136/bmjgast-2022-001092

BMJ Publishing Group Limited (BMJ) disclaims all liability and r[illegible]
placed on this supplemental material which has been supplied [illegible]

Information Leaflets Available:

1. Liver Patient Support (this one)
2. Accommodation for patients & families
3. Autoimmune Hepatitis
4. Alcoholic Liver Disease
5. Looking After Your Liver
6. Primary Biliary Cholangitis (PBC)
7. Coping With Stress
8. Primary Liver Cancer
9. You and Your Consultant
10. Primary Sclerosing Cholangitis (PSC)
11. NAFLD Lifestyle Guide
11a. Non Alcoholic Steatohepatitis
12. Liver Disease
13. Skin Care for Liver Patients
14. Diet and Liver Disease
14a. Nutrition in Liver Disease
15. Hepatitis C
16. Travel Insurance for Liver Patients
17. Hepatitis E
18. Fatigue in Liver Patients/A Patient's Journey
19. Understanding Healthcare Tests
20. Liver Cirrhosis Self Management Toolkit *
21. Exercise & Osteoporosis in Liver Patients
22. Hepatic Encephalopathy

* only from your doctor or healthcare professional

How to access LIVErNORTH information leaflets:

1. ISSUU: https://issuu.com/livernorth

2. Download from our website:
(http://www.livernorth.org.uk/pages/factsheet.htm)

3. Collect from our display boards:
(Various Hospitals & Clinics)

4. Email us: info@livernorth.org.uk

5. Phone/FAX: 0191 3702961

6. Write to us:

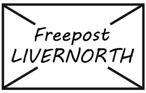

(no postage stamp necessary)

7. Facebook message us:
https://www.facebook.com/livernorth/

Yes I donate
ORGAN DONATION

Take time to talk about organ donation

Many people don't realise that their family's support is needed for organ donation to go ahead.

Information Service provided by:

LIVErNORTH

Tel & Helpline: 0191 3702961
Info@livernorth.org.uk
www.livernorth.org.uk
Registered Charity Number 108722

LIVERNORTH is a national liver patient support charity and has provided this leaflet free of charge

We have no paid employees.

Patron: George Maguire

President: Professor OFW James MA BM BCh FRCP FAMSci,

Chairman: JE Bedlington MSc MIFE MILM

Medical Advisory Committee:
Professor Quentin M Anstee BSc(Hons) MBBS PhD MRCP(UK) FRCP,
Professor David Jones OBE MA BM BCh PhD FRCP,
Professor Derek Manas FRCS BSc MBBCh Mmed (UCT) FRCSEd FCS (SA),
Dr Anand V Reddy MD, FRCP,
Professor Fiona Oakley PhD BSc,
Professor Helen Reeves BM BS BMedSci FRCP PhD,
Mr Colin Wilson MBBS FRCS PhD

NIHR non-commercial Partner

Research applications invited - download application form from website

NHS *National Institute for Health Research*

Postal address:

freepost LIVERNORTH

Find 'LIVErNORTH' on:

twitter issuu Peoples Fundraising

This leaflet is for information only. Professional, medical or other advice should be obtained before acting on anything contained in this leaflet. LIVERNORTH can accept no responsibility as a result of action taken or not taken because of the contents.
